# Dual-Directional Regulation of *Belamcanda chinensis* Extract on Ovalbumin-Induced Asthma in Guinea Pigs of Different Sexes Based on Serum Metabolomics

**DOI:** 10.1155/2022/5266350

**Published:** 2022-03-31

**Authors:** Jun Liu, Jinghe Zhu, Hong Jiang, Shiliang Zhang, Si Tang, Rui Yang, Xiaoqian Dong, Liyan Zhang

**Affiliations:** ^1^Liaoning Academy of Traditional Chinese Medicine Treatment (The Second Hospital Affiliated to Liaoning University of Traditional Chinese Medicine), Shenyang, China; ^2^College of Public Health, China Medical University, Shenyang, China; ^3^Basic Medical College of Liaoning University of Traditional Chinese Medicine, Shenyang, China

## Abstract

To explore the potential biomarkers and metabolic pathways underlying the anti-asthma effects of *Belamcanda chinensis* extract, this study established an ovalbumin-induced allergic bronchial asthma model in guinea pigs. Sixty guinea pigs were randomly divided into the blank control group, model control group, *Belamcanda chinensis* extract groups (0.8 g/kg, 1.2 g/kg, 1.6 g/kg, respectively), and dexamethasone acetate tablet group (0.5 mg/kg). Starting on the 22nd day, the drugs were administered by gavage for seven consecutive days. Serum and bronchoalveolar lavage fluid (BALF) were collected. The levels of IL-4 and IgE in the serum and IFN-*γ* and TNF-*α* in the BALF were detected by ELISA. UPLC-MS was combined with multivariate statistical analyses, including partial least squares discriminant analysis (PLS-DA), and differential metabolites between groups were identified. Metabolic pathway analysis was performed by querying the KEGG (Kyoto Encyclopedia of Genes and Genomes) online database. Female and male guinea pigs were analyzed. The results showed that compared with the model group, the IgE and IL-4 serum levels were significantly decreased in the 1.6 g/kg group, and the IFN-*γ* level in the BALF was significantly increased. The TNF-*α* level was significantly decreased in the 1.2 g/kg and 1.6 g/kg groups. There were 39 common differential metabolites among females and males, and 37 differential metabolites showed opposite regulatory trends in the serum of guinea pigs with asthma and after treatment, mainly involving 17 metabolic pathways, such as pantothenate and CoA biosynthesis, the arachidonic acid mechanism, and glycerophospholipid metabolism. *Belamcanda chinensis* extract improved OVA-induced asthma, as determined based on immune mechanisms, inflammation, nerve metabolism, and energy metabolism. The serum levels of metabolites produced by the model animals exhibited distinct sex-specific differences, and the treatment effect of *Belamcanda chinensis* extract also showed sex-specific differences and bidirectional regulation.

## 1. Introduction

*Belamcanda chinensis* is the dried root of *Belamcanda chinensis* (L.) Redoute, which was first described in *shén nóng běn cǎo jīng* and is one of the four major classical traditional Chinese medicines. The medicine is bitter and cold and targets the lung and liver meridian. The functions of the medicine are heat clearance, detoxification, and phlegm elimination, as well as benefitting the pharynx, and it is used to treat heat and poison phlegm, cough and asthma, and sore throat [1]. It has been applied in Chinese medicine for more than 2,000 years. *Belamcanda chinensis* has often been used as the principal medicine in prescriptions for bronchial asthma in both ancient and modern times, such as in “Shegan Mahuang decoction,” which is a well-known prescription for the treatment of cold-type asthma in *jīn guì yào luè* and is known as the “ancestor of prescriptions” by recent generations. Modern research has shown that *Belamcanda chinensis* contains isoflavone compounds, dicyclic triterpenoid derivatives, and phenolic compounds with anti-inflammatory properties that affect cough, phlegm, and bronchial asthma and is clinically used for the treatment of bronchial asthma, chronic pharyngitis, and cough after infection [2]. *Belamcanda chinensis* extract is the main active component of *Belamcanda chinensis* and mainly contains isoflavone compounds such as tectoridin, tectorigenin, and irigenin. Research has shown that isoflavone compounds have obvious anti-inflammatory, cough-suppressing, expectorant, and anti-asthmatic properties in vivo and effects against bronchial smooth muscle spasm activity [3].

Bronchial asthma is closely associated with a variety of cytokines, inflammatory cells, and inflammatory media. As a chronic inflammatory disease caused by joint involvement and interaction indirectly manifested as high airway reactivity, asthma has a high incidence and wide distribution, and its pathological mechanism is not clear [4]. At present, there is no specific method for the clinical treatment of bronchial asthma. Comprehensive treatment with anti-inflammatory, symptomatic, and immune drugs is the basic method, but many drugs have advantages and disadvantages. Many patients need to take drugs for a long time, and the safety of these drugs is an urgent problem to be solved [5]. Traditional Chinese medicine has become a focus of clinical attention because of its multicomponent, multitarget, and multichannel effects and low toxicity and side effects, but its complex effects cause great challenges in studies of the mechanisms underlying its effects and evaluations of its quality. Nontargeted metabolomics technology is characterized by high flux, high sensitivity, and high resolution and shows the unique advantages of integrity and systematic investigation in traditional Chinese medicine research [6]. This study used the nontarget metabolomics method to systematically analyze the changes in the levels of endogenous small molecule metabolites in the serum of guinea pigs produced during the treatment of an OVA-induced asthmatic model with *Belamcanda chinensis* extract, to explore the potential mechanism underlying the anti-asthma effects at the overall level, and to provide a research basis for the development of new *Belamcanda chinensis* preparations. To determine whether there were sex-specific differences in the asthma model and drug effects in the experimental animals, female and male guinea pigs were analyzed before and after treatment.

## 2. Materials and Methods

### 2.1. Materials

The ultralow temperature refrigerator BCD-215DK was produced by Haier Company, Qingdao. The ultrahigh liquid chromatograph used was a Waters H-Class from Waters Corporation, USA. The time-of-flight mass spectrometer used was an ACQUITY UPLC Synapt G2-S HDMS produced by Waters Corporation, USA. The column used was an ACQUITY UPLC@HSS T3, 2.5 *μ*m, 100 × 2.1 mm, from Waters Corporation, USA.

*Belamcanda chinensis* extract was prepared by the Liaoning Institute of Traditional Chinese Treatment (batch number: 20200601). Dexamethasone acetate tablets were obtained from Anhui Jintaiyang Biochemical Pharmaceutical Co. Ltd. An ELISA kit (96T) for guinea pig IL-4, IgE, IFN-*γ*, and TNF-*α* was obtained from Nanjing Jiancheng Bioengineering Institute. Methanol was purchased from Fisher Company, USA. Formic acid was also obtained from Fisher Company, USA. Ovalbumin was purchased from Sigma, Germany. Aluminum hydroxide was obtained from Tianjin Damao Chemical Reagent Factory.

### 2.2. Animals and Treatments

#### 2.2.1. Animals

This study included 60 healthy Harley SPF guinea pigs weighing 180∼220 g, among which half were females and half were males (Liaoning Changsheng Biotechnology Co. Ltd., SYCK(Liao) 2020-0001; Certificate No.: 211002300064091, Unit License No.: SYXK(Liao) 2013-0009). All animal experiments complied with the Animal Breeding and Use Guidelines of the National Institutes of Health Laboratory (8th edition, 2011). All experimental operations were approved by the Institutional Animal Care and Use Committee (IACUC) of Liaoning Qianyi Science and Technology Development Co. Ltd. (No. 2020-963, October 15, 2020). We tried to avoid causing pain in the experimental animals and to reduce the number of animals used.

Before the experiment, the animals were housed at a room temperature of 23 ± 0.5°C and relative humidity of 50% ± 5%.

#### 2.2.2. Grouping of Experimental Animals

After 7 days of adaptive feeding, 60 guinea pigs were randomly divided into 6 groups: the blank control group, model control group, 1.6 g/kg *Belamcanda chinensis* extract group, 1.2 g/kg *Belamcanda chinensis* extract group, 0.8 g/kg *Belamcanda chinensis* extract group, and positive control group. Each group included 10 guinea pigs.

#### 2.2.3. Asthma Model Establishment

Except for the blank control group, the remaining groups were intraperitoneally injected with 0.5 ml normal saline solution containing 4% OVA (ovalbumin, OVA) and 0.2 ml normal saline solution containing 2% Al(OH)_3_ on the 1st and 8th days. Starting on the 15th day, the guinea pigs were placed in a transparent closed container and challenged with 10 ml 0.5% OVA solution by aerosol inhalation for 90 s each time for seven consecutive days. The blank control group was treated with normal saline instead of OVA.

#### 2.2.4. Administration

**Figure 1:**
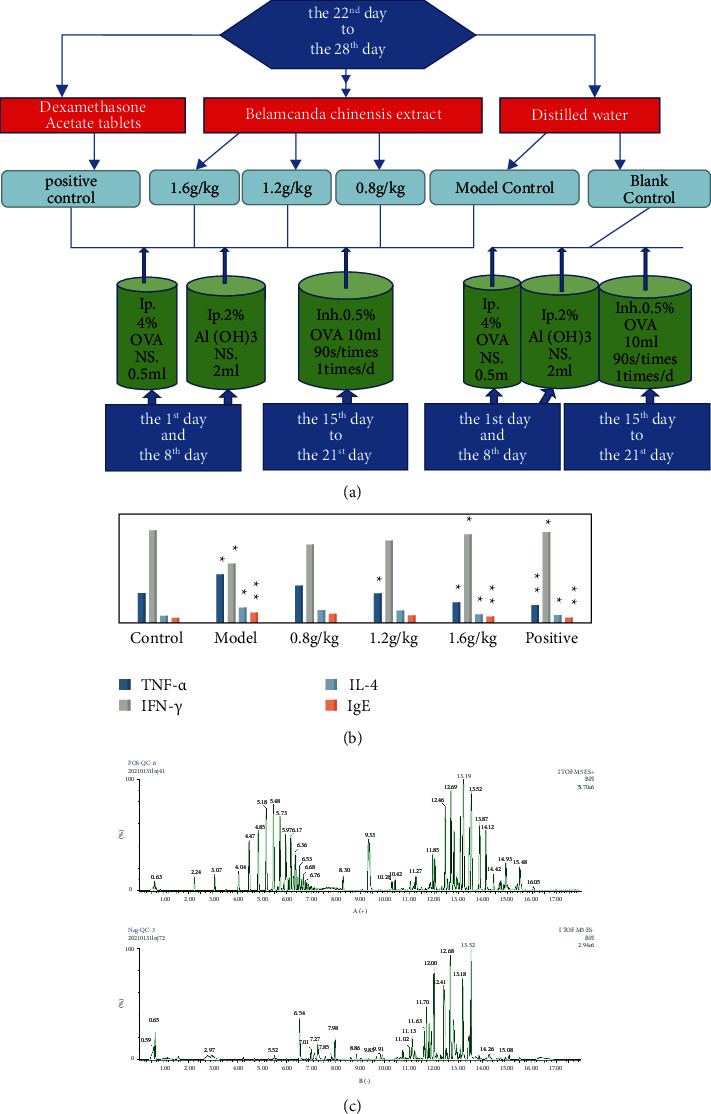
(a) Animal experimental flow chart. (b) Cytokine content detection histogram. Model group vs. control group, ^∗^*P* < 0.05 and ^∗∗^*P* < 0.01, and treatment group vs. model group, ^∗^*P* < 0.05 and ^∗∗^*P* < 0.01. (c) Base peak chromatogram (BPC) A (+) and B (−).

Starting on the 22nd day, the *Belamcanda chinensis* extract groups (0.8 g/kg, 1.2 g/kg, and 1.6 g/kg) and positive control group were administered *Belamcanda chinensis* extract by gavage. The 0.8 g/kg *Belamcanda chinensis* extract dosage was equivalent to 10 g of *Belamcanda chinensis* herbs per day for adults; the 1.2 g/kg *Belamcanda chinensis* extract dosage was equivalent to 15 g of *Belamcanda chinensis* herbs per day for adults; the 1.6 g/kg *Belamcanda chinensis* extract dosage was equivalent to 20 g of *Belamcanda chinensis* herbs per day for adults; and the positive control group was given dexamethasone acetate tablets at 0.5 mg/kg per day. The blank and model groups were given the same volume of distilled water for seven consecutive days. The animal experimental flow chart is shown in Figure 1(a).

#### 2.2.5. Specimen Collection

One hour after the last administration of 20% Urethane solution anesthesia, all guinea pigs were subjected to the following:

  Abdominal aortic blood collection:
  Five milliliters of blood was collected and centrifuged for 10 min (3,000 g, 4°C), and the serum was removed and stored in a −80°C freezer.
  Bronchoalveolar lavage (BAL):
  After blood collection, the left lung was lavaged with 4 ml of 37°C normal saline, and the lavage was repeated twice. The recovery rate was more than 80%. The collected fluid was centrifuged for 5 min (1,200 g, 4°C). The supernatant was collected and stored in a freezer at −80°C.

All animals were euthanized by carbon dioxide inhalation.

### 2.3. Detection of Cytokine Contents

To investigate the effects of asthma on experimental guinea pigs and the effect of *Belamcanda chinensis* extract on improving asthma in guinea pigs, the contents of IL-4 and IgE in the serum and TNF-*α* and IFN-*γ* in the BALF were detected by ELISA. The ELISA kit was used according to the instructions.

### 2.4. Study of Nontargeted Metabolomics Based on Liquid Chromatography/Mass Spectrometry

#### 2.4.1. Sample Preparation

Serum samples from the control, model, and 1.6 g/kg *Belamcanda chinensis* groups were thawed to room temperature in the refrigerator (4°C) and then mixed by vortexing. The addition of 50 *μ*l to 150 *μ*L methanol (−20°C, prefreezing) was used to precipitate the proteins. The samples were mixed by vortexing for 15 seconds, and then, the samples were held at −20°C for 20 min. After centrifugation for 10 min (12,000 g, 4°C), the supernatant (170 *μ*l) was collected and blown dry. A total of 120 *μ*L ultrapure water was added to redissolve the sample. Then, 100 *μ*L of the sample was transferred and ultrasonicated for 30 min. The remaining 20 *μ*L was used to mix the quality control samples. The samples were mixed and randomly injected, and the injection room temperature was maintained at 4°C.

#### 2.4.2. UPLC-QTOF-MS Analysis

  Liquid chromatographic conditions:
  Column temperature: 40°C, mobile phase A: 0.1% formic acid aqueous solution, and mobile phase B: methanol. Gradient elution parameters: 0–0.5 min, 98% A; 0.5–13 min, 98%∼1% A; 13.1–18 min, 1% A; 18.1–20 min, 1%∼98% A; and flow rate: 0.4 ml/min.
  Mass spectrometric conditions:
  Capillary voltage: 1 kV, sampling cone: 20 V, and source temperature: 120°C. Desolvation temperature: 550°C, cone gas: 150 L/h, desolvation gas: 1,000 L/h, and mass range: 50–1,200 Da.

The samples were scanned in positive and negative ion modes, and the data were collected in MassLynx 4.1 SCN 851 for peak recognition and normalization.

#### 2.4.3. Biological Information Analysis

The primary and secondary mass spectrometric data used were qualitatively and quantitatively analyzed with Progenesis QI (v2.4) and by mode identification. Compound identification analysis was performed using databases such as the Human Metabolome Database (HMDE). A multivariate statistical analysis was conducted using MetaboAnalyst 4.0. Three groups of samples (10 in each group) were divided into samples from female and male animals (6 groups with 5 samples in each). Principal component analysis (PCA) and cluster analysis were used for quality control evaluation of the QC samples, and partial least squares discriminant analysis (PLS-DA) was used to estimate the significant differences and the predictive ability of metabolic profiling between groups. The parameters R2Y (cum) and Q2 (cum) were used to assess the quality of the PLS-DA models. Values of R2Y (cum) and Q2 (cum) >0.5 and both values close to 1.0 were considered to indicate a model with outstanding predictive ability and fitness. Single-dimensional statistical analyses included fold change analysis (FCA) and Student's *t*-test, which were used to analyze significant differences in metabolites between groups. Metabolites with variable importance in the projection (VIP) >1 and *P* <0.05 were screened. The KEGG online database (Kyoto Encyclopedia of Genes and Genomes, https://www.kegg.jp/) was used for metabolic pathway enrichment analysis and pathway analysis.

### 2.5. Correlation Analysis of Differential Lipid Metabolites

Differential lipid metabolites with VIP >1.5 and *P* <0.01 were selected for clustering and correlation analysis using SPSS 17.0 to evaluate the correlations between different lipids and subclasses.

### 2.6. Confirmation of Total LPA Levels as a Biomarker of OVA-Induced Asthma in Guinea Pigs

The receiver operating characteristic (ROC) work curve of the changes in total LPA levels was constructed after the model was established, and the possibility of using the total LPA level of lipid metabolites as a potential biomarker of asthma was evaluated.

### 2.7. Total LPA Levels as a Landmark Indicator to Investigate the Effects of *Belamcanda chinensis* Extract on OVA-Induced Asthma in Guinea Pigs

ROC curves were constructed to compare the changes in the total LPA levels between the 1.6 g/kg *Belamcanda chinensis* extract group and the model group and to explore the effect of *Belamcanda chinensis* extract on the total LPA levels in the guinea pig asthma model.

### 2.8. Statistical Treatment

The data obtained in the experiment are presented as the mean ± standard error (*x* ± s.e.m.), and single-factor variance analysis (ANOVA) was used to determine the significance of differences between groups. This analysis was conducted with SPSS 17.0 by selecting the LSD (least significant difference) and SNK (Student–New Keels) tests, and *P* <0.05 indicated significant differences.

## 3. Results

### 3.1. General Characterization

During the experiment, the guinea pigs gradually developed symptoms of asthma, such as breathing with an open mouth, shortness of breath, abdominal movement, nasal involvement, sneezing and runny nose, restlessness, and scratching of the ears and nose. Compared with the guinea pigs before the experiment and the blank control group, the model guinea pigs exhibited significant differences in behavioral activities. After treatment with *Belamcanda chinensis* extract and the positive control drug, some asthma symptoms were alleviated to varying degrees in each treatment group.

### 3.2. Detection of the IL-4, IgE, IFN-*γ*, and TNF-*α* Cytokine Contents

The results showed that the serum IL-4 and IgE levels were significantly elevated in the asthmatic animals compared with the blank control animals (*P* < 0.05 and *P* < 0.01). Compared with those in the model control group, the levels of IL-4 and IgE in each *Belamcanda chinensis* extract dosage group were decreased. Among these groups, the 1.6 g/kg *Belamcanda chinensis* extract group and positive control group were compared with the model control group, and the reduction level was more significant (*P* < 0.05 and *P* < 0.01). Compared with that in the blank control group, the IFN-*γ* level in the BALF of the model control group was significantly decreased (*P* < 0.05), and the levels of TNF-*α* were significantly increased (*P* < 0.05). Compared with the model group, the 1.6 g/kg *Belamcanda chinensis* extract group and positive control group exhibited significantly increased levels of IFN-*γ* (*P* < 0.05). In the 1.2 g/kg *Belamcanda chinensis* extract group, 1.6 g/kg *Belamcanda chinensis* extract group, and positive control group, the levels of TNF-*α* were significantly decreased (*P* < 0.05 and *P* < 0.01). The histogram is shown in Figure 1(b).

### 3.3. Analysis by Untargeted Metabolomics

The UPLC-QTOF-MS method was used for base peak chromatography (BPC) detection, as shown in Figures 1(c) (A and B).

#### 3.3.1. Bioinformatics Analysis

The results showed that the three groups compared were not fully distinguished. The female and male samples were also separated and compared, and the sex comparison showed significant differences among the groups.

**Figure 2:**
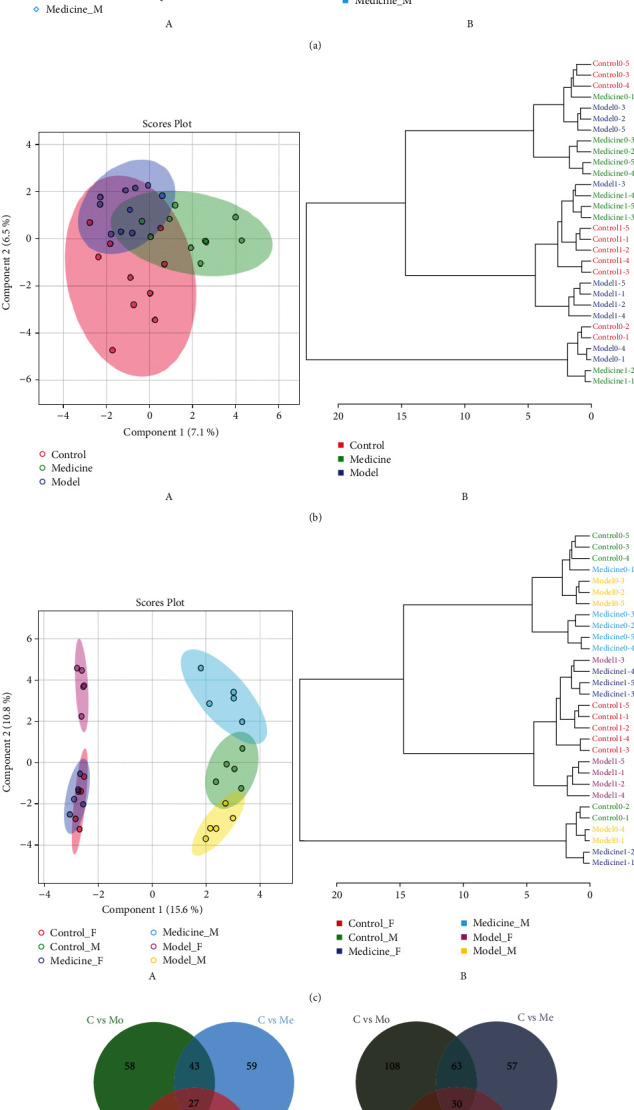
(a) Control, model, and treatment groups: PCA diagram of all samples and the QC samples (A) and cluster analysis diagram (B). (b) Control, model, and treatment groups (*n* = 10 per group): PLS-DA diagram (A) and cluster analysis diagram (B). (c) Control, model, and treatment groups (*n* = 5 per group): PLS-DA (A) and cluster diagram (B). (d) A. Venn diagram cross-analysis of males and B. Venn diagram cross-analysis of females.

Mass spectrometry data were obtained for all samples in the control, model, and 1.6 g/kg *Belamcanda chinensis* extract groups and the QC samples. PCA and cluster analysis showed that the QC samples were closely clustered together and distributed among the other samples, indicating good quality control, as shown in Figure 2(a).

The three groups (10 samples from each group) were compared and were not fully distinguished (Figure 2(b)). The comparison by sex (6 groups with 5 samples per group) showed significant differences among the sexes (Figure 2(c)). In the samples from female animals (A), the control and treated samples clustered together and were completely distinct from the model samples. In the samples from male animals (B), there were differences among the three groups. The model and treatment groups differed, and the control group was located between these two groups. The preliminary results showed that *Belamcanda chinensis* extract had certain sex-specific effects on asthma, and the treatment effect on female animals with asthma was stronger.

When the female and male samples were separated and compared, the first 25 metabolites with large VIPs could be significantly distinguished by cluster thermograms.

**Figure 3:**
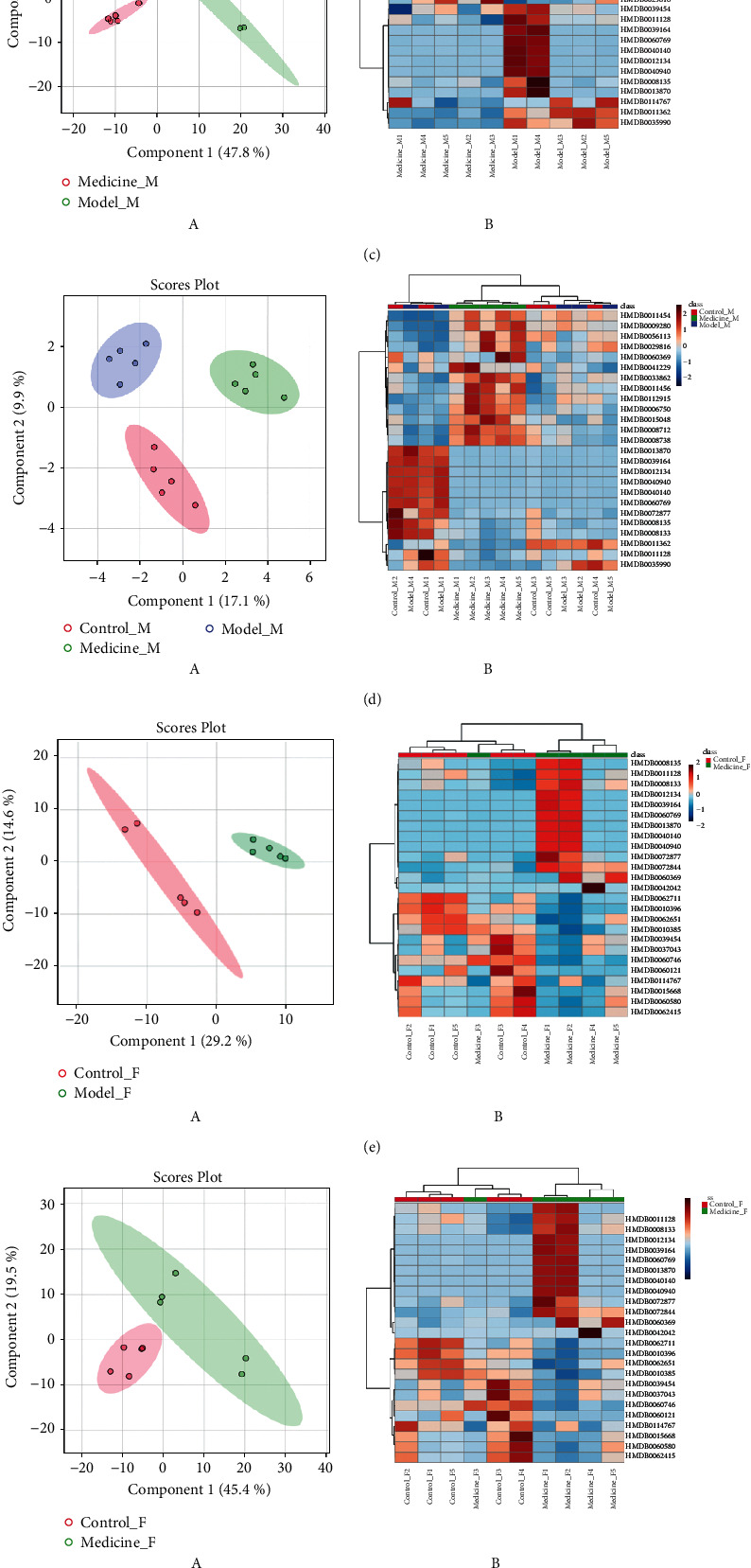
(a) Control vs. model groups: PLS-DA diagram of male samples (A) and cluster heat map (B). (b) Control vs. treatment groups: PLS-DA diagram of male samples (A) and cluster heat map (B). (c) Model vs. treatment groups: PLS-DA diagram of male samples (A) and cluster heat map (B). (d) Control vs. model vs. treatment groups: PLS-DA diagram of male samples (A) and cluster heat map (B). (e) Control vs. model groups: PLS-DA diagram of female samples (A) and cluster heat map (B). (f) Control vs. model groups: PLS-DA diagram of female samples (A) and cluster heat map (B). (g) Model vs. treatment groups: PLS-DA diagram of the female samples (A) and cluster heatmap (B). (h) Control vs. model vs. treatment groups: PLS-DA diagram of female samples (A) and cluster heat map (B).

Male
    Control versus model: the two groups were fully distinguished, as shown in Figure 3(a).
    Control versus treatment: the two groups could also be distinguished, but the distance was relatively close, as shown in Figure 3(b).
    Model versus treatment: the two groups could also be distinguished, as shown in Figure 3(c).
    Control versus model versus treatment groups: the three groups were distinguished. The control and treatment samples were distributed on the same side, as shown in Figure 3(d).
Female
    Control versus model: the two groups were fully distinguished, as shown in Figure 3(e).
    Control versus treatment: the two groups could also be distinguished, but the distance was relatively close, as shown in Figure 3(f).
    Model versus treatment: the two groups were fully distinguished, as shown in Figure 3(g).
    Control versus model versus treatment groups: the first 25 metabolites with larger VIPs in the cluster heat maps were selected and failed to fully cluster; however, the control group was close to the treatment group, distributed on the same side, and far from the model group, as shown in Figure 3(h).

The results showed that the model group was significantly different from the control and treatment groups. There were also differences between the control and treatment groups, but their relative positions were close, which indicated that *Belamcanda chinensis* extract could significantly improve the serum differential metabolites induced by ovalbumin, and the effects of *Belamcanda chinensis* extract were sex-specific.

#### 3.3.2. Differential Metabolite Screening

Analyses were conducted separately for female and male samples. The male analysis groups were as follows: C1 – control vs. model; C2 – control vs. treatment; and C3 – model vs. treatment. The comparative analyses of female samples were as follows: C3 – control vs. model; C5 – control vs. treatment; and C6 – model vs. treatment. The results were as follows.

C1 identified 213 different metabolites, including 3b,12b-dihydroxy-5b-cholanoic acid, 6-dehydrotestosterone glucuronide, and 10,11-dihydro-20-trihydroxyleukotriene B4. C2 identified 259 differential metabolites. C3 identified 370 differential metabolites, such as cortisol, dolichol phosphate, and 10,11-dihydro-20-trihydroxyleukotriene B4. C4 identified 390 differential metabolites, including sphingosine 1-phosphate, leukotriene D4, and succinmer. C5 identified 197 differential metabolites. C6 identified 363 differential metabolites, including 12-ketodeoxycholic acid, N-sulfo-D-glucosamine, and tyrosyl-serine.

Three groups of males and females were compared to identify differential metabolites via Venn cross-analysis, as shown in Figure 2(d) A and B. There were 27 common differential metabolites in males and 30 common differential metabolites in females.

Males: C1 and C3 were cross-analyzed and had 112 common differential metabolites, and 27 differential metabolites common in C2 were excluded. We preliminarily determined that 85 of the differential metabolites produced after the establishment of asthma in male animals were significantly improved after treatment.

Females: C4 and C6 were used for cross-analysis. There were 218 common differential metabolites, and 30 differential metabolites common in C5 were excluded. It was preliminarily determined that 188 of the differential metabolites produced after the establishment of asthma in female animals were significantly improved after treatment.

There were 32 common lipid differential metabolites between C1 and C3, meaning that of the 40 lipid differential metabolites produced by male animals with asthma, the levels of 32 were significantly restored after treatment with *Belamcanda chinensis* extract. The major subclasses of CDP-DG 11s, CL 2s, PE 6s, PA 3s, LPE 2s, and so on in the C3 total LPC were decreased; however, the changes were not significant. The total LPA levels were more significantly decreased. C4 and C6 shared 57 differential lipid metabolites; that is, 57 of 90 differential lipid metabolites produced by female animals with asthma showed significantly restored levels after treatment with *Belamcanda chinensis* extract. The main subclasses of CDP-DG 14s, TG 3s, PC 7s, PA 1s, LPC 2s, and so on, the total LPC, and the total LPA were decreased significantly in C6.

Common differential metabolites between males and females included 39 differential metabolites among C1, C3, C4, and C6, such as leukotriene B4 ethanolamide, CDP-DG (18:1(9Z)/18:0), all-trans-report phenphate, PC (22:5 (7Z, 10Z, 13Z, 16Z, 19Z)/22:4 (7Z, 10Z, 13Z, 16Z)), PE (P-18:1(9Z)/14:0), and so on. These differential metabolites showed common significant differences between males and females after the establishment of asthma and showed significant trends of regression after treatment. Thirty-seven metabolites changed in opposite directions in the sera of the different sexes, and the regulatory effects of *Belamcanda chinensis* extract on differential serum metabolites in guinea pigs of different sexes were also opposite. For example, for the differential metabolite all-trans-heptaprenyl diphosphate, the metabolic trends were as follows: C1, decreased; C3, increased; C4, increased; and C6, decreased. Differences in amino acid metabolism-related metabolites, such as serylserine and threoninyl-serine, also exhibited different trends in female and male guinea pigs.

The results of differential metabolite analyses showed that the numbers and types of differential serum metabolites produced between female and male guinea pigs were quite different, and there were significant sex differences in the regulation of serum metabolites in the asthma model and *Belamcanda chinensis* extract groups. The effects on the model were sex-specific, and the treatment and regulatory effects of *Belamcanda chinensis* extract on the model were also bidirectional, with the effects on female guinea pigs being more significant.

#### 3.3.3. Metabolic Pathway Analysis

**Figure 4:**
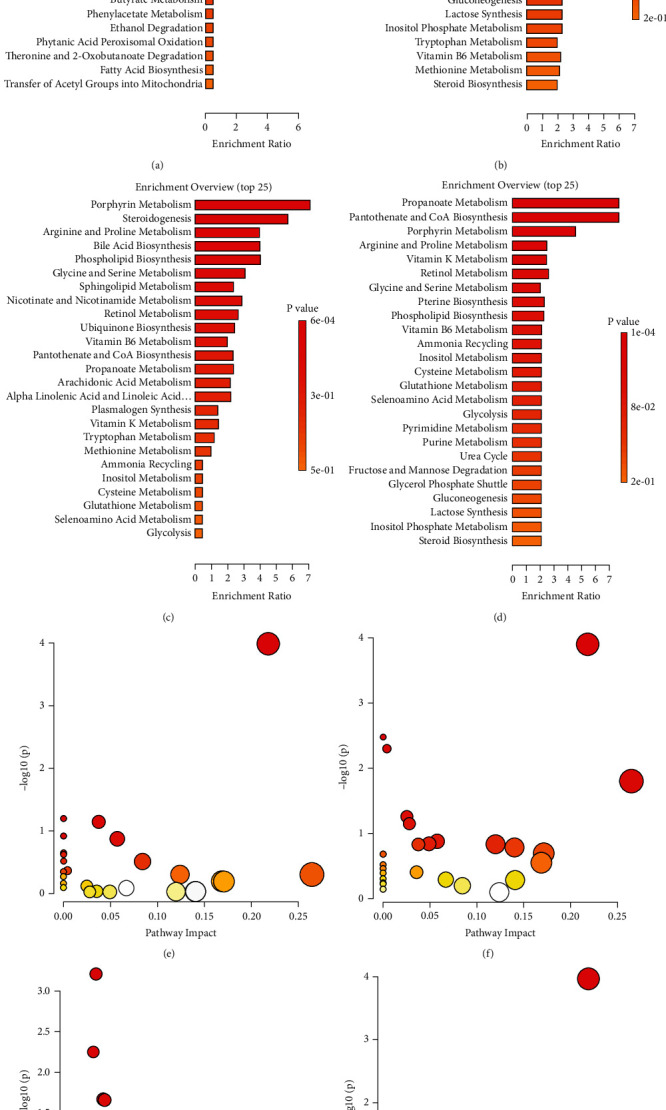
Metabolic pathway enrichment analysis diagram: (a) C1, (b) C3, (c) C4, and (d) C6. Pathway topology analysis diagram: (e) C1, (f) C3, (g) C4, and (h) C6.

The differential metabolites in C1, C3, C4, and C6 were analyzed by KEGG enrichment analysis. According to the order of Holm *P* values from small to large, the top 25 pathways were analyzed based on their enrichment ratios, as shown in Figures 4(a)–4(d).

Through the topological analysis of pathways, as shown in Figures 4(e)–4(h), 17 potential target pathways with impact values >0.003 were identified. These pathways mainly included glycerophospholipid metabolism, pantothenate and CoA biosynthesis, sphingolipid metabolism, fatty acid degradation, pentose and glucuronate interconversions, terpenoid backbone biosynthesis, glycosylphosphatidylinositol (GPI) anchor biosynthesis, and so on.

There were significant differences in the metabolic pathways identified in the female and male guinea pigs during the establishment of the model (C1 vs. C4). There were 91 common differential metabolites between the sexes. Among the top 25 pathways identified in the enrichment analysis, 6 were common, and 19 were different. There were also certain differences after the treatment of the guinea pig models with *Belamcanda chinensis* extract (C3 vs. C6). The top 25 pathways identified by enrichment analysis included 20 common pathways and 5 different pathways. However, compared with the differences in the models, the mechanism of *Belamcanda chinensis* extract treatment was relatively less sex-specific.

### 3.4. Analysis of Differential Lipid Correlations among C1, C3, C4, and C6

**Figure 5:**
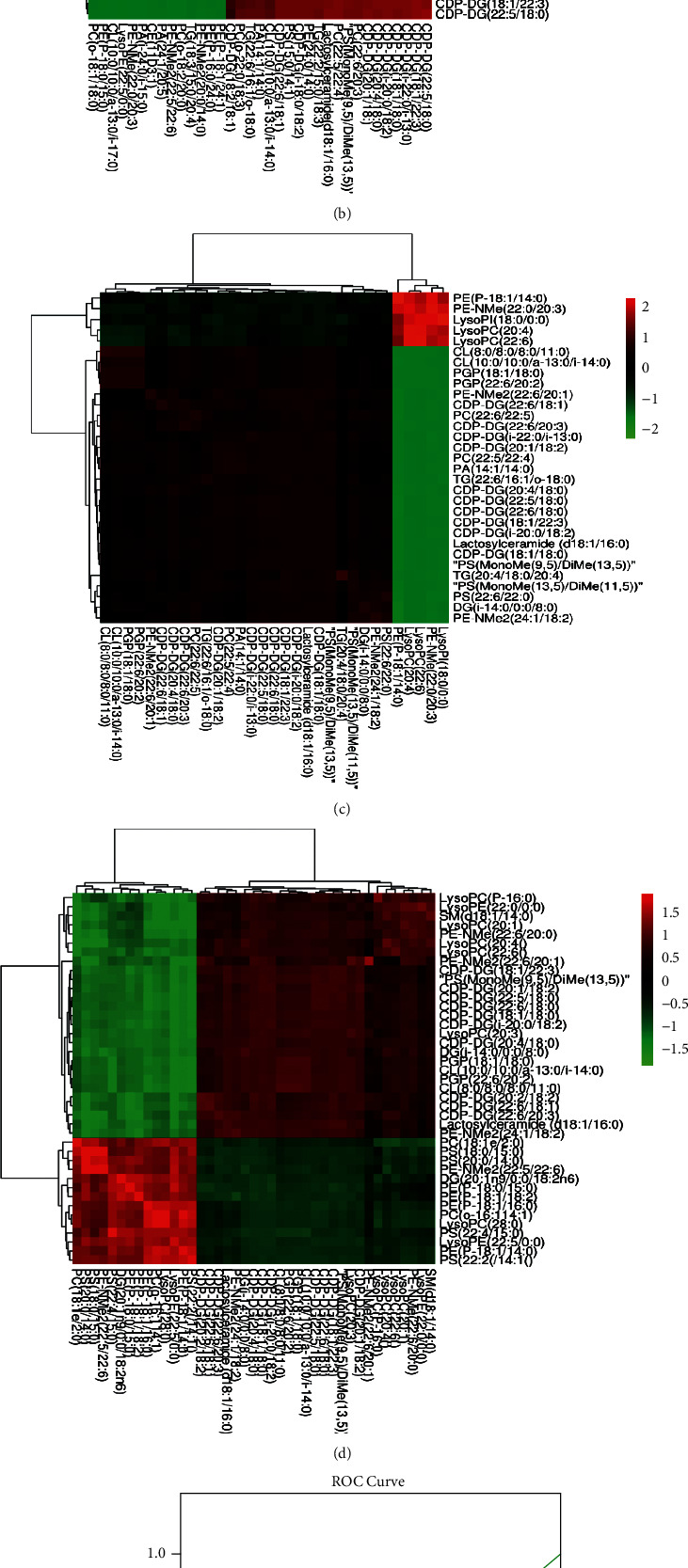
Diagram of differential lipid correlation analysis: (a) C1, (b) C3, (c) C4, and (d) C6. (e) ROC working curve of total LPA levels in the model and control groups. (f) ROC working curve of total LPA levels between the model and treatment groups.

There is a mutual conversion relationship between lipid metabolites in vivo. Differential lipid metabolites with VIP >1.5 and *P* < 0.01 were selected from C1, C3, C4, and C6 and analyzed by SPSS 17.0 to evaluate the correlations between the levels of different lipids and lipid subclasses in various groups. We analyzed 18 significantly differential lipids in C1, including CDP-DG, PE (24:1/15:0), and PS (MonoMe (9,5)/DiMe (13,5)); 35 significantly differential lipids in C3, including lactosylceramide (d18:1/16:0), PS (MonoMe (9,5)/DiMe (13,5)), PE (P-18:0/15:0), and PC; 31 significantly differential lipids in C4, including CDP-DG, PS, PA, and PE; and 41 significantly differential lipids in C6, including lactosylceramide (d18:1/16:0), CDP-DG, PS, DG, and PE. Generally, the correlation strength of variables is determined by the absolute value of the correlation coefficient as follows: 0.8–1.0, extremely strong correlation; 0.6–0.8, strong correlation; 0.4–0.6, moderate correlation; 0.2–0.4, weak correlation; and 0.0–0.2, extremely weak correlation or no correlation. The correlations were divided into three grades, with 0 indicating uncorrelated, ±1 indicating a strong correlation, and ±2 indicating a very strong correlation. The heat map is shown in Figures 5(a)–5(d).

The analysis results showed the following:

1. The correlation between the CDP-DG subclasses was strong, and most of the subclasses showed strong correlations (|*r*| > 0.8). There were strong correlations between the CDP-DG subclasses and most other subclasses.
2. There were strong correlations between the molecules in the CL and PGP subclasses.
3. C1: CDP-DG showed strong correlations with PE (24:1/15:0), LysoPE (22:5/0:0), LysoPE (22:6/0:0), and PS (MonoMe (9,5)/DiMe (13,5)) (|*r*| > 0.9).
4. C3: Lactosylceramide (d18:1/16:0), CDP-DG, PS (MonoMe (9,5)/DiMe (13,5)), PE (P-18:0/15:0), PC, CL (10:0/10:0/a-13:0/i-17:0) (|*r*| > 0.8), CDP-DG and PE (24:0/14:0), LysoPE (22:5/0:0), PC (22:5/22:4), PC (22:6/20:3), PS (MonoMe (9,5)/DiMe (13,5)), and CL showed strong correlations.
5. C4: Lactosylceramide (d18:1/16:0) was strongly associated with CDP-DG, PS, PA, and PE; PC was correlated with PA; and PS, PE, and LysoPC were strongly associated with CL, PGP, and LysoPI (18:0/0:0).
6. C6: Lactosylceramide (d18:1/16:0) was strongly associated with CDP-DG, PS, DG, and PE; LPE (22:5/0:0) was strongly associated with CDP-DG, LysoPC (20:3), and PS (MonoMe (9,5)/DiMe)/DiMe (13,5)); and PGP was strongly associated with DG (20:1n9/0:0/18:2n6), PS (MonoMe (9,5)/DiMe (13,5)), and so on.

These results indicated that interconversions between molecules of the same subclass and between different subclasses of lipids may have occurred.

### 3.5. Confirmation of Total LPA as a Biomarker of OVA-Induced Asthma in Guinea Pigs

The area under the ROC curve (AUC) of the total LPA values in the model control group compared with the blank control group was 0.72, with good specificity and good sensitivity. The total LPA value of lipid metabolites may be a potential biomarker of asthma, as shown in Figure 5(e).

### 3.6. Total LPA as a Landmark Indicator for Investigating the Effects of *Belamcanda chinensis* Extract on an OVA-Induced Guinea Pig Asthma Model

The ROC working curve of the total LPA abundance values in the model control group compared with that of the treatment group was constructed. The area under the ROC curve (AUC) was 0.78 (*P* = 0.034), with *P* < 0.05 and good specificity and sensitivity. *Belamcanda chinensis* extract had significant effects on the total lipid LPA levels in the OVA-induced guinea pig asthma model, as shown in Figure 5(f).

## 4. Discussion

Previous studies have shown that *Belamcanda chinensis* extract mainly contains isoflavones, including tectoridin, tectorigenin, iristectorigenin A, iristectorigenin B, irigenin, irisflorentin, and dichotomitin, and has significant anti-inflammatory, cough, anti-viral, anti-bacterial, anti-oxidant, estrogen-like, anti-tumor, and other pharmacological effects [7, 8]. Tectorigenin, irigenin, and irisflorentin have been reported to have anti-inflammatory effects. Tectorigenin levels are negatively correlated with interleukin-4 (IL-4) levels in the alveoli and serum; irigenin levels are also negatively correlated with IL-4 levels in the serum; and tectorigenin significantly reduces the content of leukotriene C4 (LTC4) [9]. Tamura [10] found that tectorigenin inhibits the activity of the high-affinity receptor Fc*ε*RI on immunoglobulin E (IgE), which is an important molecule in human mast cells that causes allergic reactions, indicating that tectorigenin has an anti-allergic effect.

According to the Chinese Pharmacopoeia 2020 edition [1], the dosage of *B. chinensis* is 3–10 g per day for adults. Combined with the preliminary research results, the three *Belamcanda chinensis* extract dosages of 0.8 g/kg, 1.2 g/kg, and 1.6 g/kg were selected, which were equivalent to the adult *Belamcanda chinensis* extract dosages of 10 g, 15 g, and 20 g per day, respectively.

An increase in IgE levels in vivo indicates immune dysfunction. Antigens activate T cells and B lymphocytes to synthesize a large amount of IgE antibodies, and these cells produce cytokines, such as IL-4 and IL-13, which have mainly anti-inflammatory effects [11]. TNF-*α* promotes the secretion of related inflammatory mediators (such as IL-4) to participate in the occurrence and development of asthma and is one of the important reasons for the persistence of airway inflammation. An imbalance between the Th1 and Th2 cell subsets is the central link in the pathogenesis of allergic asthma. Antigenic stimulation decreases the proportion of the Th1 cell subgroup, significantly decreases the IFN-*γ* concentration, increases the proportion of the Th2 cell subgroup, and significantly increases the IL-4 concentration in the serum [12].

Pantetheine, which is a common metabolite between male and female animals, is involved in the pantothenate and CoA biosynthesis metabolic pathways. In these pathways, the synthesis of upstream metabolites is related to the glycolysis pathway. Asthma is characterized by airway hyperresponsiveness and the contraction of airway smooth muscle, which easily lead to a decrease in inhaled oxygen and hypoxia and may lead to an increase in anaerobic glycolysis. Pantetheine is used to synthesize pantetheine 4′-phosphate downstream, and pantetheine 4′-phosphate is the precursor in the synthesis of CoA within organisms. CoA is mainly involved in fatty acid and pyruvate metabolism. In the synthesis and oxidation of fatty acids and the tricarboxylic acid cycle, the pyruvate acid oxidation process plays very important roles. Asthma leads to breathing difficulties; thus, more energy is needed for breathing. Tricarboxylic acid circulation acts as the hub of sugar, fat, and amino acid metabolism in the body, providing the basis for cell energy production. Pantetheine exerts different regulatory effects in female and male guinea pigs. We speculate that the expression of different enzymes caused by sex hormones and other factors may be directly or indirectly regulated in the respective pathways, leading to differences in metabolic directions.

Both the female and male models involved the leukotriene of arachidonic acid metabolites, leukotriene D4 (LTD4). LTD4 is mainly involved in arachidonic acid metabolism. Arachidonic acid metabolism is closely related to pathological processes such as inflammation, allergies, and immunity. LTD4 is a metabolite of arachidonic acid. The slow-reacting substance (SAS-A) in an allergic reaction is a mixture of LTD4, LTC4, and LTE4 and has a strong effect on bronchial smooth muscle contraction. LTD4 also increases capillary permeability. In this experiment, LTD4 was increased in C1 and C4, but the effects of treatment with *Belamcanda chinensis* extract on the model were sex-specific. The drug significantly improved the female guinea pig model (C6 significant reduction) but had almost no effect on the male model (C3 showed no downtrend). The specific regulatory mechanism needs to be further studied.

LTB4 is also one of the metabolites of arachidonic acid that can cause neutrophils and eosinophils to migrate and produce inflammatory infiltration. In this study, we found a significant increase in 10,11-dihydro-20-trihydroxy-leukotriene B4 levels among the differential metabolites in male model guinea pigs (C1), and 10,11-dihydro-20-trihydroxy-leukotriene B4 may be an endogenous metabolite of LTB4 [13]. These elevated levels may be due to changes in the LTB4 levels in vivo. However, the changes in the 10,11-dihydro-20-trihydroxy-leukotriene B4 levels in female model guinea pigs (C4) were not significant. *Belamcanda chinensis* extract significantly decreased the metabolite level in male guinea pigs with asthma (C3) and did not significantly improve the level in female guinea pigs with asthma (C6). Based on these results combined with those regarding IgE and IL-4 levels, in the arachidonic acid metabolic pathway of the OVA-induced guinea pig asthma model, due to sex differences, different inflammatory mediators were changed, and *Belamcanda chinensis* extract also regulated these changes accordingly.

Another metabolite associated with arachidonic acid is leukotriene B4 ethanolamide. Douglas McHugh demonstrated that [14] LTB4 ethanolamide could act as an antagonist of the LTB4 receptor in the lungs and significantly antagonize the chemotaxis of LTB4, increasing the Ca^2+^ concentration in rTRPV1-transfected CHO cells; thus, it acts as an agonist of the TRPV1 receptor but is less effective than capsaicin [14]. The TRPV1 channel protein is closely related to the pathological processes of inflammation and can be activated/sensitized by various immune response products. Inflammatory factors can act on a receptor coupled to the G protein or indirectly activate TRPV1 by activating phospholipase C (PLC) or A_2_ (PLA_2_), inducing the release of arachidonic acid metabolites, such as anandamide, through the tyrosine kinase pathway [15]. LTB4 ethanolamide may be an endogenous metabolite of 5-lipoxygenase of anandamide [15], but the associated metabolic pathway in the body is not clear. In our study, sex-specific differences in LTB4 ethanolamide occurred as follows: C1, increased, and C4, decreased. *Belamcanda chinensis* extract also bidirectionally regulated this metabolite, and whether different metabolic pathways were affected needs to be further confirmed.

Regarding lipid subclass metabolites, the total LPA levels in female and male guinea pigs increased in C1 and C4, while the total LPA levels decreased significantly after treatment in C3 and C6. Preliminarily, *Belamcanda chinensis* extract may play an intervention role by significantly lowering the total serum LPA level in guinea pigs with OVA-induced asthma. Studies have found that the level of total LPA in BALF is increased in asthmatic patients. In mouse models of allergic airway inflammation and in asthma patients after the allergic intervention, LPA (C22:5 and C22:6) was selectively synthesized, and polyunsaturated hemolytic phospholipid acid (LPA) was considered a potential asthma biomarker [16]. LPA has multiple effects on airway and lung inflammation, suggesting its importance in the physiological physiology of asthma [17]. LPA can increase the secretion of interleukin-8 (IL-8), promote PGE2 release, and regulate the secretion of IL-3 to induce neutrophils to aggravate inflammation [18].

In female guinea pig serum, the lactosylceramide (d18:1/16:0) level was also different, and cerebroside B, 3-O-sulfogalactosylceramide (d18:1/20:0), ganglioside GM3 (d18:1/14:0), and so on and metabolic pathways such as sphingolipid metabolism were altered.

Sphingolipids and their metabolites participate in and regulate airway inflammation, airway remodeling, and airway hyperresponsiveness. Studies have found that in guinea pig asthma models [19], higher ceramide (Cer) synthetase activity and ceramide levels in the airway cortex led to coughing, breathing difficulties, and severe bronchoconstriction in guinea pigs, indicating that ceramide is involved in allergic asthma and airway inflammation. C1P is produced when ceramide is phosphorylated by ceramide kinase, and a decrease in C1P in the plasma membrane and increase in C1P in the Golgi complex can stimulate the release of cytoplasmic phospholipase A2*α* and induce the production of proinflammatory arachidonic acid [20]. In this study, 3-O-sulfogalactosylceramide (d18:1/20:0) and lactosylceramide (d18:1/16:0) were significantly different in female guinea pigs in C4 and C6.

In the guinea pig asthma model, airway reactivity is associated with the level of ganglioside in the trachea. In the acute period of the high airway response, the increased accumulation of ganglioside species in the trachea mainly includes polysialic acid ganglioside [21]. Significant changes in ganglioside GM3 (d18:1/14:0) and ganglioside GA2 (d18:1/9Z-18:1) in the female guinea pig model indicated that ganglioside was also involved in the development of bronchial asthma. *Belamcanda chinensis* extract had a significant effect on the regulation of ganglioside GM3 (d18:1/14:0).

The results showed that *Belamcanda chinensis* extract may have interfered with the OVA-induced asthma guinea pig model through a neuroregulation mechanism, which may have affected related metabolites, such as ceramide, ganglioside, and cerebroside, and these effects were more significant in female guinea pigs with asthma.

Studies have shown that serum metabolism is significantly different in asthma patients of different sexes, and lipid metabolism is significantly associated with sex [22]. The biotransformation of the liver is affected by sex factors, and the expression levels of bioinvertase in males and females are different [23]. Studies have shown that the bioinvertase induction effects of some drugs are different in male and female rats, which may be caused by the different hormone levels between male and female rats [24]. In addition, males generally have a significantly higher basal metabolic rate than females. Whether these factors are related to the sex differences in the model and *Belamcanda chinensis* extract effects in this study needs further research.

Female and male animals had differential levels of the metabolite dolichol phosphate, which exhibited the same trend in guinea pigs of different sexes; that is, it was downregulated after asthma establishment and upregulated after *Belamcanda chinensis* extract treatment. Dolichol phosphate is primarily associated with N-glycan biosynthesis and closely related to the biosynthesis of N-linked polysaccharides in glycoproteins in vivo. Immunoglobulin G (IgG) is an N-linked glycoprotein, and changes in its levels are inseparable from the occurrence and development of allergic asthma; thus, it is an important marker of allergic asthma [11]. *Belamcanda chinensis* extract may regulate the metabolism level of dolichol phosphate and other metabolites in the body and interfere with the expression of glycoproteins related to immune mechanisms to play a role in the treatment of allergic asthma.

In conclusion, guinea pigs exhibited symptoms of asthma after stimulation with 0.5% OVA solution, resulting in significant increases in factors such as IgE and IL-4 in the serum. The IFN-*γ* level was significantly reduced, and a significant increase in the TNF-*α* level was observed in the BALF. Inflammatory factors, such as serum LTD4, and the lipid total LPA level increased, and the dolichol phosphate level decreased. Significant changes in the levels of metabolites, such as lactosylceramide, cerebroside B, pantetheine, all-trans-heptaprenyl diphosphate, and different degrees of change and transformation of the lipid subclasses PA, PC, LPC, and so on were observed. These effects led to bronchospasm, airflow obstruction, airway inflammation, airway hyperresponsiveness, and so on. *Belamcanda chinensis* extract improved some of the related symptoms and the levels of IL-4 and IgE in the serum and IFN-*γ* and TNF-*α* in the BALF, reduced the total LPA level, and increased the dolichol phosphate level. In female guinea pigs, the level of LTD4 was significantly downregulated, while, in male guinea pigs, the level of ceramide was significantly upregulated, and most of the metabolic regulation trends exhibited sex-specific differences. According to KEGG metabolic pathway analysis, *Belamcanda chinensis* extract treatment regulated immune-inflammatory pathways, such as arachidonic acid metabolism, and neuroregulatory mechanisms, such as neuroactive ligand-receptor interactions and sphingolipid metabolism, as well as glycerophospholipid metabolism and the phospholipase D signaling pathway. These effects were associated with energy metabolic pathways such as glycolysis via N-glycan biosynthesis and pantothenate and CoA biosynthesis.

**Figure 6:**
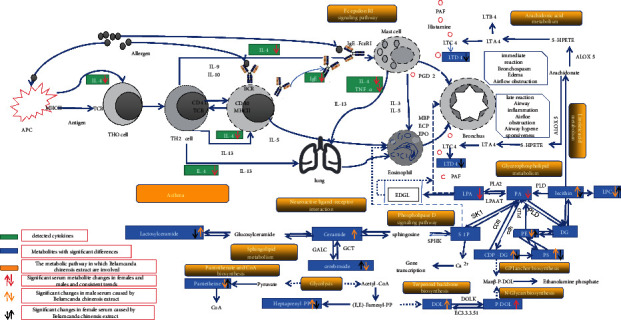
Mechanistic diagram of the effects of *Belamcanda chinensis* extract on OVA-induced guinea pig asthma.

A mechanistic diagram of the effects of *Belamcanda chinensis* extract on OVA-induced guinea pig asthma is shown in Figure 6.

The common metabolic pathways among female and male animals also included bile acid synthesis, steroid hormone biosynthesis, vitamin metabolic pathway, and so on. In addition to these pathways, female animals also showed the involvement of arginine and proline metabolism and porphyrin metabolism.

## 5. Conclusion

This study found that OVA-induced asthma in guinea pigs mainly affects arachidonic acid metabolism, glycerophospholipid metabolism, pantothenate and CoA biosynthesis, sphingolipid metabolism, neuroactive ligand-receptor interactions, fatty acid biosynthesis, N-glycan biosynthesis, glycolysis, and other metabolic pathways. There were significant sex-specific differences in the regulation of serum metabolites in this model. There were differences in the types and quantities of metabolites between female and male guinea pigs with asthma, resulting in differences in metabolic pathways and metabolic mechanisms. In general, female guinea pigs were more sensitive to the establishment of asthma than male guinea pigs, and the regulatory effects on the vast majority of metabolites in guinea pigs of different sexes were opposite. The treatment effects of *Belamcanda chinensis* extract also showed sex-specific differences and bidirectional regulation of metabolite levels.

## Data Availability

All data used during this study are available from the corresponding author upon request.

## Supplementary Materials

Supplementary Materials Table 1: male and female common differential metabolites. Table 2: numbers of differential metabolites. Table 3: pathway enrichment analysis. Table 4: metabolic pathway analysis. Table 5: IL-4, IgE, IFN-*γ*, and TNF-*α* contents.
