# Supplementary material for: Dual-Directional Regulation of Belamcanda chinensis Extract on Ovalbumin-Induced Asthma in Guinea Pigs of Different Sexes Based on Serum Metabolomics
Source: Evid Based Complement Alternat Med. 2022 Mar 31;2022:5266350. doi: 10.1155/2022/5266350 (PMC8991378; doi:10.1155/2022/5266350)
Supplement: Supplementary Materials — Table 1: male and female common differential metabolites. Table 2: numbers of differential metabolites. Table 3: pathway enrichment analysis. Table 4: metabolic pathway analysis. Table 5: IL-4, IgE, IFN-γ, and TNF-α contents. [file 5266350.f1.doc]

**Supporting Information**

**Effect of *Belamcanda chinensis* Extract on OVA-Induced Asthma in Guinea Pigs Based on Serum Metabolomics**

Jun Liu^1^, Jinghe Zhu^1^, Hong Jiang^2^，Shiliang Zhang^1^, Si Tang^1^, Rui Yang^1^, Xiaoqian Dong^1^, Liyan Zhang^3^

^1^Liaoning Academy of Traditional Chinese Medicine Treatment,Shenyang,China

^2^College of Public Health, China Medical University,Shenyang,China

^3^Basic Medical College of Liaoning University of Traditional Chinese Treatment,Shenyang,China

Corresponding author: Dr. Jun Liu

Liaoning Academy of Traditional Chinese Medicine Treatment

No. 60, Huanghe North Street

Huanggu District

Shenyang,Liaoning

China

E-mail address: zijun.999@126.com

Phone: (+86)18940158949

**Contents**

Table 1 Male and Female common differential metabolites

| No. | Metabolites | Change  (C1) | Change  (C3) | Chang  （C4） | change  （C6） |
| --- | --- | --- | --- | --- | --- |
| 1 | Leukotriene B4 ethanolamide | up | down | down | up |
| 2 | Pantetheine | up | down | down | up |
| 3 | Docosahexaenoyl Ethanolamide | up | down | down | up |
| 4 | 2-Methyl-1,4-naphthalenediol bis(dihydrogen phosphate) | up | down | up | down |
| 5 | N-(4-aminobutyl)-3-(4-hydroxyphenyl)prop-2-enimidic acid | up | down | down | up |
| 6 | All-trans-heptaprenyl diphosphate | down | up | up | down |
| 7 | Threoninyl-Serine | down | up | up | down |
| 8 | (3beta,17alpha,23S)-17,23-Epoxy-3,28,29-trihydroxy-27-norlanost-8-en-24-one | down | up | up | down |
| 9 | exo-Dehydrochalepin | down | up | up | down |
| 10 | Dolichol phosphate | down | up | down | up |
| 11 | 2-Chloromaleylacetate | down | up | up | down |
| 12 | 17-alpha-ethinyl estradiol | down | up | up | down |
| 13 | 3,4,5-trihydroxy-6-(3,4,5-trihydroxyphenoxy)oxane-2-carboxylic acid | down | up | up | down |
| 14 | 15-(3-methylbut-2-en-1-yl)-8,17-dioxatetracyclo-heptadeca-2,4,6,11(16),12,14-hexaene-3,5,14-triol | down | up | up | down |
| 15 | CDP-DG(18:1(9Z)/18:0) | down | up | up | down |
| 16 | CDP-DG(18:1(9Z)/22:3(10Z,13Z,16Z)) | down | up | up | down |
| 17 | CDP-DG(20:1(11Z)/18:2(9Z,12Z)) | down | up | up | down |
| 18 | CDP-DG(20:2(11Z,14Z)/18:2(9Z,12Z)) | down | up | up | down |
| 19 | CDP-DG(20:4(5Z,8Z,11Z,14Z)/18:0) | down | up | up | down |
| 20 | CDP-DG(22:5(7Z,10Z,13Z,16Z,19Z)/18:0) | down | up | up | down |
| 21 | CDP-DG(22:6(4Z,7Z,10Z,13Z,16Z,19Z)/18:0) | down | up | up | down |
| 22 | CDP-DG(22:6(4Z,7Z,10Z,13Z,16Z,19Z)/18:1(9Z)) | down | up | up | down |
| 23 | CDP-DG(i-18:0/18:2(9Z,11Z)) | down | up | up | down |
| 24 | CDP-DG(i-20:0/18:2(9Z,11Z)) | down | up | up | down |
| 25 | CDP-DG(i-22:0/i-13:0) | down | up | up | down |
| 26 | PC(22:5(7Z,10Z,13Z,16Z,19Z)/22:4(7Z,10Z,13Z,16Z)) | down | up | up | down |
| 27 | LPC(20:4(8Z,11Z,14Z,17Z)) | up | down | down | up |
| 28 | PE(P-18:1(9Z)/14:0) | up | down | down | up |
| 29 | LPE(22:5(7Z,10Z,13Z,16Z,19Z)/0:0) | up | down | down | up |
| 30 | PGP(18:1(9Z)/18:0) | down | up | up | down |
| 31 | PGP(22:6(4Z,7Z,10Z,13Z,16Z,19Z)/20:2(11Z,14Z)) | down | up | up | down |
| 32 | PGP(i-12:0/i-24:0) | down | up | up | down |
| 33 | CL(8:0/8:0/8:0/11:0) | down | up | up | down |
| 34 | CL(10:0/10:0/a-13:0/i-14:0) | down | up | up | down |
| 35 | TG(22:2(13Z,16Z)/15:0/18:3(9Z,12Z,15Z)) | down | up | up | down |
| 36 | TG(22:6(4Z,7Z,10Z,13Z,16Z,19Z)/16:1(9Z)/o-18:0) | down | up | up | down |
| 37 | PS(MonoMe(13,5)/DiMe(11,5)) | down | up | up | down |
| 38 | PS(MonoMe(9,5)/DiMe(13,5)) | down | up | up | down |
| 39 | PE-NMe(22:0/20:3(8Z,11Z,14Z)) | up | down | down | up |

Table 2 the number of differential metabolites

| sexs | **groups** | differential metabolites |
| --- | --- | --- |
| Male | C1 | 40 |
|  | C3 | 141 |
|  | C1 and C3 | 32 |
| Female | C4 | 90 |
|  | C6 | 109 |
|  | C4 and C6 | 57 |

Table 3 Pathways Enrichment Analysis

| **N0.** | **groups** | **Pathways Enrichment** |
| --- | --- | --- |
| 1 | C1 | | Pantothenate and CoA Biosynthesis | | --- | | Propanoate Metabolism | | Nicotinate and Nicotinamide Metabolism | | Bile Acid Biosynthesis | | Vitamin K Metabolism | | Retinol Metabolism | | Beta-Alanine Metabolism | | Caffeine Metabolism | | Oxidation of Branched Chain Fatty Acids | | Valine, Leucine and Isoleucine Degradation | | Lysine Degradation | | Glycerolipid Metabolism | | Amino Sugar Metabolism | | Fatty acid Metabolism | | Beta Oxidation of Very Long Chain Fatty Acids | | Fatty Acid Elongation In Mitochondria | | Citric Acid Cycle | | Ketone Body Metabolism | | Butyrate Metabolism | | Phenylacetate Metabolism | | Ethanol Degradation | | Phytanic Acid Peroxisomal Oxidation | | Threonine and 2-Oxobutanoate Degradation | | Fatty Acid Biosynthesis | | Transfer of Acetyl Groups into Mitochondria | |
| 2 | C3 | | Propanoate Metabolism | | --- | | Pantothenate and CoA Biosynthesis | | Bile Acid Biosynthesis | | Nicotinate and Nicotinamide Metabolism | | Phospholipid Biosynthesis | | Steroidogenesis | | Porphyrin Metabolism | | Ammonia Recycling | | Inositol Metabolism | | Cysteine Metabolism | | Glutathione Metabolism | | Selenoamino Acid Metabolism | | Glycolysis | | Pyrimidine Metabolism | | Purine Metabolism | | Urea Cycle | | Fructose and Mannose Degradation | | Glycerol Phosphate Shuttle | | Gluconeogenesis | | Lactose Synthesis | | Inositol Phosphate Metabolism | | Tryptophan Metabolism | | Vitamin B6 Metabolism | | Methionine Metabolism | | Steroid Biosynthesis | |
| 3 | C4 | | Porphyrin Metabolism | | --- | | Steroidogenesis | | Arginine and Proline Metabolism | | Bile Acid Biosynthesis | | Phospholipid Biosynthesis | | Glycine and Serine Metabolism | | Sphingolipid Metabolism | | Nicotinate and Nicotinamide Metabolism | | Retinol Metabolism | | Ubiquinone Biosynthesis | | Vitamin B6 Metabolism | | Pantothenate and CoA Biosynthesis | | Propanoate Metabolism | | Arachidonic Acid Metabolism | | Alpha Linolenic Acid and Linoleic Acid Metabolism | | Plasmalogen Synthesis | | Vitamin K Metabolism | | Tryptophan Metabolism | | Methionine Metabolism | | Ammonia Recycling | | Inositol Metabolism | | Cysteine Metabolism | | Glutathione Metabolism | | Selenoamino Acid Metabolism | | Glycolysis | |
| 4 | C6 | | Propanoate Metabolism | | --- | | Pantothenate and CoA Biosynthesis | | Porphyrin Metabolism | | Arginine and Proline Metabolism | | Vitamin K Metabolism | | Retinol Metabolism | | Glycine and Serine Metabolism | | Pterine Biosynthesis | | Phospholipid Biosynthesis | | Vitamin B6 Metabolism | | Ammonia Recycling | | Inositol Metabolism | | Cysteine Metabolism | | Glutathione Metabolism | | Selenoamino Acid Metabolism | | Glycolysis | | Pyrimidine Metabolism | | Purine Metabolism | | Urea Cycle | | Fructose and Mannose Degradation | | Glycerol Phosphate Shuttle | | Gluconeogenesis | | Lactose Synthesis | | Inositol Phosphate Metabolism | | Steroid Biosynthesis | |

Table 4 Metabolic pathway analysis

| N0. | Pathway Name | Match Status | p | -log(p) | Holm p | FDR | Impact |
| --- | --- | --- | --- | --- | --- | --- | --- |
| 1 | Glycerophospholipid metabolism | 4/36 | 0.50017 | 0.30088 | 1.0 | 0.94742 | 0.26445 |
| 2 | Pantothenate and CoA biosynthesis | 2/19 | 1.0258E-4 | 3.989 | 0.0027696 | 0.0027696 | 0.21786 |
| 3 | Tryptophan metabolism | 4/41 | 0.64698 | 0.18911 | 1.0 | 0.94742 | 0.17085 |
| 4 | Sphingolipid metabolism | 6/21 | 0.63572 | 0.19673 | 1.0 | 0.94742 | 0.16836 |
| 5 | Pentose and glucuronate interconversions | 1/18 | 0.94742 | 0.023458 | 1.0 | 0.94742 | 0.14062 |
| 6 | Terpenoid backbone biosynthesis | 1/18 | 0.93357 | 0.029851 | 1.0 | 0.94742 | 0.13968 |
| 7 | Fatty acid degradation | 1/39 | 0.50331 | 0.29816 | 1.0 | 0.94742 | 0.12404 |
| 8 | Cysteine and methionine metabolism | 4/33 | 0.94427 | 0.024905 | 1.0 | 0.94742 | 0.11966 |
| 9 | Ether lipid metabolism | 3/20 | 0.3098 | 0.50891 | 1.0 | 0.83647 | 0.08434 |
| 10 | Glycine, serine and threonine metabolism | 3/34 | 0.83567 | 0.077967 | 1.0 | 0.94742 | 0.06662 |
| 11 | N-Glycan biosynthesis | 1/41 | 0.13527 | 0.86881 | 1.0 | 0.73044 | 0.05732 |
| 12 | Vitamin B6 metabolism | 1/9 | 0.94174 | 0.02607 | 1.0 | 0.94742 | 0.04902 |
| 13 | Pyrimidine metabolism | 1/39 | 0.071608 | 1.145 | 1.0 | 0.64447 | 0.03727 |
| 14 | Arginine and proline metabolism | 2/38 | 0.91953 | 0.036432 | 1.0 | 0.94742 | 0.03558 |
| 15 | Porphyrin and chlorophyll metabolism | 1/30 | 0.93902 | 0.027326 | 1.0 | 0.94742 | 0.02799 |
| 16 | Steroid hormone biosynthesis | 4/77 | 0.7537 | 0.1228 | 1.0 | 0.94742 | 0.02515 |
| 17 | Glycosylphosphatidylinositol (GPI)-anchor biosynthesis | 1/14 | 0.43616 | 0.36035 | 1.0 | 0.94742 | 0.00399 |

Table 5 IL-4、IgE、IFN-γ、TNF-α Content

| Groups | **Serum** | | **BALF** | |
| --- | --- | --- | --- | --- |
|  | **IL-4(ng/L)** | **IgE（U/ml）** | **IFN-γ(ng/L)** | TNF-α**(ng/L)** |
| Bland Control group | 12.660±2.298 | 8.979±1.576 | 1.701E2±2.981E1 | 54.671±9.131 |
| Model Control group | 28.122±3.673^*^ | 18.652±2.470^**^ | 1.087E2±9.253^*^ | 89.238±10.146^*^ |
| 0.8g/kg group | 23.357±5.42 | 16.160±2.254 | 1.440E2±1.258E1 | 68.275±16.85 |
| 1.2g/kg group | 22.415±6.327 | 13.690±2.087 | 1.512E2±1.067E1 | 53.899±14.605^*^ |
| 1.6g/kg group | 15.510±1.508^*^ | 11.167±2.050^*^ | 1.626E2±1.178E1^*^ | 37.383±6.290^**^ |
| positive control group | 14.095±4.654^*^ | 9.750±0.902^**^ | 1.668E2±1.735E1^*^ | 32.677±5.040^**^ |

Model Vs Control，* p＜0.05；** p＜0.01；The extract of *Belamcanda chinensis* 0.8 g/kg、1.2 g/kg、1.6 g/kg groups and positive control group vs the model control group，* p＜0.05；** p＜0.01。
